# Simultaneous Genetically Detection of *Streptococcus* *pyogenes*, *Streptococcus* *pneumoniae* and *Haemophilus* *influenzae* in Patients with Treatment-Resistant Respiratory Infection

**DOI:** 10.30699/ijp.2023.1991067.3075

**Published:** 2023-07-16

**Authors:** Farzad Mohammadi Ebli, Zoheir Heshmatipour, Khadijeh Daneshjou, Seyed Davar Siadat

**Affiliations:** 1 *Department of Biology, Science and Research Branch, Islamic Azad University, Tehran, Iran*; 2 *Department of Microbiology, Tonekabon Branch, Islamic Azad University, Tonekabon, Iran*; 3 *Department of Pediatrics, Imam Khomeini Hospital, School of Medicine, Tehran University of Medical Sciences, Tehran, Iran*; 4 *Department of Mycobacteriology and Pulmonary Research, Microbiology Research Center, Pasteur Institute of Iran, Tehran, Iran*

**Keywords:** Gene, Multiplex PCR, Pathogen, Sputum

## Abstract

**Background & Objective:**

*Streptococcus* *pneumoniae*, *Haemophilus* *influenzae* and *Streptococcus* *pyogenes* are among the most important causes of infection in human. Inventing rapid methods to identify these species can help in providing appropriate and effective treatment options. Therefore, the current study aimed to develop a multiplex touch-down PCR method to identify rapidly the aforementioned species patients' sputum samples, simultaneously.

**Methods:**

A total of 50 sputum samples of patients with respiratory infections resistant to treatment were collected. After DNA extraction and primer design, the complete capsule (CAP) region II, capsular polysaccharide biosynthesis (cpsA) and the structural regulator of transcription (spy) genes were amplified for detecting *H. influenzae*, *S. pneumoniae* and *S. pyogenes *by multiplex touch-down PCR.

**Results:**

Among 50 samples prepared from patients with different diseases, 27 samples were positive for amplified genes. The frequency of presence of pathogens in the collected samples included 14% *H. influenzae*, 20% *S. pneumoniae* and 20% *S. pyogenes*. Also, in some patients, the simultaneous presence of two or three pathogens were observed.

**Conclusion:**

In general, it can be concluded that the PCR touchdown method developed in the present study is an effective and fast method for the simultaneous identification of* H. influenzae*, *S. pneumoniae* and *S. pyogenes* pathogens in clinical samples of patients.

## Introduction

*Streptococcus* *pneumoniae* is the main cause of pneumonia, and it is one of the important causes of meningitis, bacteremia, sepsis, otitis media and sinusitis (1). Classically, the diagnosis of this microorganism is obtained from its proper growth from the appropriate sample. This bacterium has been one of the most important human pathogens known in the last 100 years, which has caused many illnesses and deaths (2). *S. pneumoniae* is easily colonized in the nasopharyngeal canal and causes a wide range of asymptomatic and mild diseases to serious respiratory infections and invasive infections such as meningitis (3). *Streptococcus* *pyogenes* also causes a wide range of non-fatal surface diseases such as impetigo, pharyngitis and fatal diseases such as streptococcal toxic shock syndrome (STSS) (4). *Haemophilus* sp. also causes a wide range of diseases in humans such as respiratory diseases (5). The dominant species of this bacterium is *Haemophilus* *influenzae*, which is mostly isolated in the oral cavity and pharynx, but is absent in the nasal cavity. The non-capsulated strain of this bacterium, biotype III and II, is mainly present in the pharynx of healthy children (6). All the three aforementioned bacteria are present as normal flora in the upper respiratory system and can become pathogenic when a person has a weakened immune system.

Respiratory system diseases have different causes, but they have common clinical and pathophysiological characteristics. The bacteria present in the respiratory system enter this area in different ways; some of them enter the lower air system through inhalation of air and another group enter the lower air system through microaspiration of mucous secretions from the oral cavity and upper air system (7). In a healthy person, the innate defenses in the respiratory system generally prevent infection. Innate immune mechanisms include mucociliary clearance, engulfment of foreign substances by alveolar macrophages, and opsonization or inhibition of bacteria by soluble liquid components on the airway surface (8). 

Diagnosing the mentioned bacteria through traditional methods such as culture is very cheap and facilitates the identification of antibiotic resistance. But these methods are time-consuming and the possibility of creating false results disrupts the treatment process and lead to choosing inappropriate antibiotics. Therefore, it is vital to develop new methods that are very sensitive, accurate and fast (9). Recently, more specialized culture-based approaches allowed the detection of most organisms associated with the human body. However, the cost of this effort is very unfortunate both in terms of time and money (10). The development of molecular approaches that are able to identify many species in parallel by examining the genetic content or microbiome provides a more comprehensive view of microbial communities (11). Several indirect methods, including fingerprinting [denaturing gradient gel electrophoresis (DGGE), terminal restriction fragment length polymorphisms (T-RFLP), temperature-time gradient gel electrophoresis (TTGE)], hybridization (clonal hybridization, microarray), and PCR Multiple touch has been used to investigate the microbiota in clinical samples (12). The aim of this study was to develop a multiplex PCR method to detect *H. influenzae*, *S. pneumoniae* and *S. pyogenes* in clinical sputum samples.

## Material and Methods

**Clinical Samples**

Sputum samples were obtained from 50 patients hospitalized in the ICU department of Pars Hospital in Tehran, Ieran, between March 2018 and August 2019. All clinical data including gender, age and the underling diseases were obtained and recorded. After examining the samples by gram staining and confirming the presence of less than 5 epithelial cells, the samples were treated with mycolysin solution. In this phase, the samples were incubated for 90 min at 37°C and then stored at -70°C for further investigation. Written consent was obtained from all the patients to enter the study. The researchers adhered to the Declaration of Helsinki during the implementation of the experiment and sampling.

**DNA Extraction and Primer Design**

Microbial DNA extraction kit was used for DNA extraction (Qiagen, Hilden, Germany). The quality and quantity of extracted DNA were confirmed by NanoDrop™ (Thermofisher, Waltham, Massachusetts, United States) and gel electrophoresis, respectively. 

The sequences of primers used for amplifying the complete capsule (*CAP*) region II, capsular polysaccharide biosynthesis (*cpsA*) and the structural regulator of transcription (*spy*) genes are given in Table 1.

**Table 1:** The sequences of primers used for amplification of *spy*, *cpsA* and *cap* genes

Species	Genes	Amplicon size (bp)	Primer sequence (5'-3')	Reference
*S. pyogenes*	*spy*	407	F:AAAGACCGCCTTAACCACCT R:TGGCAAGGTAAACTTCTAAAGCA	Luo, Y.C.,* et al. *2012
*S. pneumoniae*	*cpsA*	177	F:AGTGGTAACTGCCTTAGTCCTA R:GTGGCGTTGTGGTCAAGAG	Luo, Y.C.,* et al. *2012
*H. influinzae*	*cap*	653	F:ATGTTAGATCGTGCGGATACTC R:GCGAGGAACAGAACCATCAG	Luo, Y.C.,* et al. *2012

**Touchdown PCR**

The reaction mixture included 25 μL of mastermix (RED amplicon), 1.6 μL of *spy* primer, 1.4 μL of *cpsA* primer, 1 μL of *cap* primer, 9 μL of DNA, and 12 μL of deionized water, making a final volume of 50 μL. 

The PCR time-temperature program consisted of one cycle of 94°C for 7 min, 20 cycles of 94°C for 30 s, 61°C for 30 s, and 72°C for 30 s, 15 cycles of 95°C for 30 s, 55°C for 30 s and 72°C for 30 s, and final a cycle of 72°C for 7 min.

At the end, the PCR products were sent to Pishgam Cop. (IRAN) to determine the sequence of nucleotides, and the results were analyzed on the NCBI website and the Blast program, and finally the desired sequence was compared with other bacteria in terms of homology.

## Results

**Clinical Data**

Out of the 50 examined patients, all of them were suffering from treatment-resistant nosocomial infections, and in terms of gender, 30 were men (60%) and 20 were women (40%). These patients were in the age range of 21 to 90 years. The most common underling diseases were diarrhea and COPD (each 12%). Also, pneumoniae, influenza and rectal cancer (each 10%) were prevalence in patients. Of the 50 samples, 40 (80%) were sputum, 7 (17%) were Bronchoscopy and bronchoalveolar lavage (BAL) and 3 (6%) were tracheal tube (Table 2).

**Table 2:** Clinical data of the patients enrolled in current study

Characteristics	N (%)
Gender	50
Male	30(60)
Female	20(40)
Underling diseases	
ARDS	2(4)
Embolism	2(4)
Bronchiectasis	4(8)
COPD	6(12)
Pneumoniae	5(10)
Asthma	4(8)
Influenzae	5(10)
Rectal cancer	5(10)
Tuberculosis	4(8)
Femoral fracture	2(4)
Sepsis	1(2)
Diarrhea	6(12)
Gastric cancer	3(6)
Pleurisy	1(2)
Samples	
Sputum	40(80)
Bronchoscopy and Bronchoalveolar Lavage (BAL)	7(14)
Tracheal tube	3(6)

**Molecular Identification of Pathogens**

**Fig. 1:**
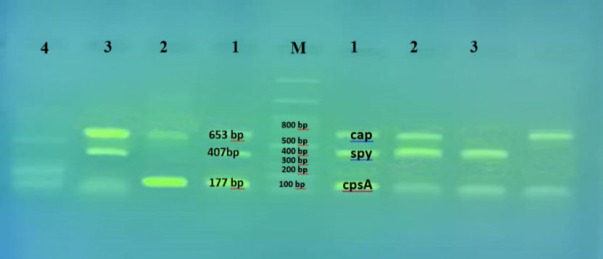
The amplifications of the complete capsule (*CAP*) region II, capsular polysaccharide biosynthesis (*cpsA*) and the structural regulator of transcription (*spy*) genes in clinical samples by touchdown multiplex PCR.

The multiplex touchdown PCR reveled 27 positive cases, in which *H. influenzae* was detected in 7 cases (14%), *S. pneumoniae* in 10 cases (20%) and *S. pyogenes* in 10 cases (20%). In 5 samples, *H. influenzae* and *S. pyogenes* were detected simultaneously. *H. influenzae* and *S. pneumoniae* were seen simultaneously in 6 samples. Finally, *S. pneumoniae* and *S. pyogenes* were detected in 5 patients, simultaneously (Figure 1). 

*H. influenzae*, *S. pneumoniae* and *S. pyogenes *frequencies in clinical samples based underling diseases are given in Table 3.

Lane 1:* H. influenzae *ATCC® 9006TM (Hib),* S. pneumoniae *ATCC® 36059TM;* S. pyogenes *ATCC® 19615TM; Lane 2 & 3:* H. influenzae*,* S. pneumoniae*,* S. pyogenes* clinical samples; Lane 4: Negative control

**Table 3:** The detections of *H. influenzae*, *S. pneumoniae* and *S. pyogenes *in clinical samples based underling diseases

PCR result	Total	ARDS	Embolism	Bronchitis	COPD	Pneumoniae	Asthma	Flu	Rectal	TB	Femor	Sepsis	Diarrhea	Gastric	Pleurisy
*[H. influenzae*	7			1	1	1	1	2		1					
*S. pneumoniae*	10			1	1	4	2	2							
*S. pyogenes*	10			1	3		2	1		2		1			
*H. influenzae* *+* *S. pyogenes*	5			1	1		1	1		1					
*H. influenzae* *+* *S. pneumoniae*	6			1	1	1	1	2							
*S. pneumoniae* *+* *S. pyogenes*	5			1	1		2	1							
Total	43														

## Discussion

Traditional methods such as culture and serology are not enough to investigate and diagnose the respiratory system microbiota. It is very difficult to quickly diagnose these bacteria based on phenotypic characteristics, so fast and modern diagnostic methods are needed (13). Today, it is known that the sensitivity and specificity of culture-based methods for detecting microbiota are very low, so methods based on high sensitivity and rapid detection, such as molecular methods, are very useful for detecting bacteria in the respiratory system microbiota (14). In the present study, both the conventional PCR method and the multiplex method were used to detect *S. pneumoniae*, *S. pyogenes* and *H. influenzae*. The results showed that the identification of microbiota using the multiplex method was faster than the conventional PCR method. In the research conducted by Maleki* et al.* (15) in Iran, it was shown that molecular methods such as multiplex PCR were the most effective in detecting the microbiota of the respiratory system. In another study in England (16), the detection of *S. pneumoniae*, *S. pyogenes*, and *H. influenzae* using molecular methods was much more efficient. Many studies have proven higher sensitivity and accuracy and less time of the multiplex method in identifying bacteria than other methods such as conventional PCR and sample culture. Similar to the findings of the present study, in a study conducted by Gillis* et al. *(17), it was observed that conventional PCR has a very low sensitivity in the detection and isolation of *S. pneumoniae* from the nasopharyngeal swab sample of pneumonia patients. This difference in identification can be due to the difference in the type of sample that was used in the present study. In the multiplex touch-down method, the sensitivity of detecting *S. pneumoniae* was lower than *H. influenzae*, which can be attributed to the presence of the *autolysin A* gene in *Streptococcus*, reducing the sensitivity of *S. pneumoniae* detection (18). In the study conducted by Shakib and Zolfaghari (19), it was found that Real time PCR technique has 100% sensitivity in detection *S. pneumoniae* present in sputum. Fan* et al. *in China (20) reported that the sensitivity of the multiplex PCR method in identifying *Haemophilus* species for *omp6* and *bexA* genes were 100 and 99.8%, respectively. In our study, *H. influenzae*, *S. pneumoniae* and *S. pyogenes* were detected in 14%, 20% and 20% of samples, respectively. In the study of Aydemir *et al.* in Turkey (21), *S. pneumoniae*, *H. influenzae* and *S. pyogenes* were identified in 15.2, 12.7 and 14.7 %, respectively. In another study in England (22), *H. influenzae* (40.2%) was more isolated than *S. pneumoniae* (35.6%). However, very few studies in Iran have investigated microbiota bacteria related to respiratory infections. In a study conducted by Naderi* et al. *(23), the highest isolation rate was related to *S. pneumoniae* (24.4%). The reported frequency was higher than in our studies for *S. pneumoniae*. In the research conducted by Temesgen* et al. *in Ethiopia (24), which was conducted using culture methods and biochemical tests, the rate of identification of *S. pneumoniae* was higher than in the present study (35.9) and the rate of isolation of 2 other bacteria*, H. influenzae* and *S. pyogenes* ( 8.4 and 6.9%, respectively).

According to the research done in relation to the molecular diagnosis of pathogenic agents, in this research we tried to improve the PCR method and use new and innovative techniques to save costs and consumables in the laboratory routines and rapid diagnosis of pathogens to speed up the process of diagnosis, prevention and treatment. As in this experiment, we also checked the quality of bacteria detection with the classic multiplex PCR method, and the result was that it took more time and money to reach the appropriate protocol, and unwanted products and non-specific bands were created during amplification. In similar projects that were carried out in different parts of the world, they also found that the unwanted production during gene amplification is reduced with the multiplex touch-down PCR method compared to the simple multiplex method. In addition, by simultaneously performing the detection of bacteria using the traditional method such as biochemical tests and serological tests found that almost both diagnostic methods were similar in terms of correct identification, while the multiplex touch-down PCR method is much lower in terms of time and cost, and has higher accuracy.

## Conclusion

Early identification of infectious agents in the respiratory system can reduce complications and prevent overuse of antimicrobial drugs. Conventional and old methods such as culture and serology are not always sufficient to detect the microbiota of the lower respiratory tract. Therefore, new diagnostic methods are needed. In this study, we designed and implemented the multiplex touch-down PCR method to evaluate their clinical effectiveness (25, 26). This study describes the development and evaluation of a single-tube, three-target, multiplex touch PCR assay that can simultaneously detect *S. pneumoniae*, *H. influenzae*, and *S. pyogenes* directly on clinical sputum samples. The *CpsA* gene has recently been identified as a target for *S. pneumoniae* specific detection and has been found to be able to differentiate *S. pneumoniae* from *Streptococcus pseudopneumoniae* and was chosen in our study (27, 28). Targets for PCR-based detection of *H. influenzae* are relatively rare in published articles. We identified that the amplifications of complete capsule (*CAP*) region II, capsular polysaccharide biosynthesis (*cpsA*) and the structural regulator of transcription (*spy*) genes are effective in identification of *S. pneumoniae*, *H. influenzae*, and *S. pyogenes* pathogens in clinical samples.

## Conflict of Interest

There is no conflict of interest among authors.

## References

[1] Loughran AJ, Orihuela CJ, Tuomanen EI (2019). Streptococcus pneumoniae: Invasion and Inflammation. Microbiol Spectr..

[2] Walker CLF, Rudan I, Liu L, Nair H, Theodoratou E, Bhutta ZA (2013). Global burden of childhood pneumonia and diarrhoea. Lancet..

[3] Brandileone MC, Casagrande ST, Guerra MLS, Zanella RC, Andrade ASS, Fabio JD (2006). Increase in numbers of beta-lactam-resistant invasive Streptococcus pneumoniae in Brazil and the impact of conjugate vaccine coverage. J Med Microbiol..

[4] Avire NJ, Whiley H, Ross K (2021). A Review of Streptococcus pyogenes: Public Health Risk Factors, Prevention and Control. Pathogens [Internet]..

[5] Wen S, Feng D, Chen D, Yang L, Xu Z (2020). Molecular epidemiology and evolution of Haemophilus influenzae. Infect Genet Evol..

[6] Takeuchi N, Ohkusu M, Hoshino T, Yamamoto S, Segawa S, Murata S (2021). Emergence of Haemophilus influenzae with low susceptibility to quinolones isolated from pediatric patients in Japan. J Infect Chemother..

[7] Marik PE (2001). Aspiration pneumonitis and aspiration pneumonia. N Engl J Med..

[8] Hartl D, Tirouvanziam R, Laval J, Greene CM, Habiel D, Sharma L (2018). Innate Immunity of the Lung: From Basic Mechanisms to Translational Medicine. J Innate Immun..

[9] Boyles T, Wasserman S (2015). Diagnosis of bacterial infection. S Afr Med J..

[10] Trotter AJ, Aydin A, Strinden MJ, O'Grady J (2019). Recent and emerging technologies for the rapid diagnosis of infection and antimicrobial resistance. Curr Opin Microbiol..

[11] Gerace E, Mancuso G, Midiri A, Poidomani S, Zummo S, Biondo C (2022). Recent Advances in the Use of Molecular Methods for the Diagnosis of Bacterial Infections. Pathogens..

[12] Ferrera I, Balagué V, Voolstra CR, Aranda M, Bayer T, Abed RM (2014). Molecular methods for biofilms. Biofouling Methods..

[13] Goodarzi NN, Pourmand M, Rajabpour M, Arfaatabar M, Mosadegh M, Mohamad SS (2020). Frequency of Mycoplasma pneumoniae Legionella pneumophila and Chlamydia spp among patients with atypical pneumonia in Tehran. New Microbes New Infect..

[14] Lim HJ, Kang ER, Park MY, Kim BK, Kim MJ, Jung S (2021). Development of a multiplex real-time PCR assay for the simultaneous detection of four bacterial pathogens causing pneumonia. PLoS One..

[15] Maleki A, Mansournia F, Ghafourian S, Taherikalani M, Pakzad I, Mohammadi J (2020). Rapid and direct molecular detection of Streptococcus pneumoniae and Haemophilus influenzae isolated in oropharynx and nasal cavity of children. New Microbes New Infect..

[16] Thors V, Morales-Aza B, Pidwill G, Vipond I, Muir P, Finn A (2016). Population density profiles of nasopharyngeal carriage of 5 bacterial species in pre-school children measured using quantitative PCR offer potential insights into the dynamics of transmission. Hum Vaccin Immunother..

[17] Gillis HD, Lang ALS, ElSherif M, Martin I, Hatchette TF, McNeil SA (2017). Assessing the diagnostic accuracy of PCR-based detection of Streptococcus pneumoniae from nasopharyngeal swabs collected for viral studies in Canadian adults hospitalised with community-acquired pneumonia: a Serious Outcomes Surveillance (SOS) Network of the Canadian Immunization Research (CIRN) study. BMJ Open..

[18] Bjarnason A, Lindh M, Westin J, Andersson LM, Baldursson O, Kristinsson KG (2017). Utility of oropharyngeal real-time PCR for S pneumoniae and H influenzae for diagnosis of pneumonia in adults. Eur J Clin Microbiol Infect Dis..

[19] Shakib P, Zolfaghari MR (2021). Detection of Streptococcus pneumoniae in sputum samples by real-time PCR. Anti-Infect Agents..

[20] Fan X, Liu X, Ji L, Cai D, Jiang J, Zhu J (2018). Epidemiological analysis and rapid detection by one-step multiplex PCR assay of Haemophilus influenzae in children with respiratory tract infections in Zhejiang Province, China. BMC Infect Dis..

[21] Aydemir O, Aydemir Y, Ozdemir M (2014). The role of multiplex PCR test in identification of bacterial pathogens in lower respiratory tract infections. Pak J Med Sci..

[22] Gadsby NJ, Russell CD, McHugh MP, Mark H, Conway Morris A, Laurenson IF (2016). Comprehensive Molecular Testing for Respiratory Pathogens in Community-Acquired Pneumonia. Clin Infect Dis..

[23] Naderi H, Sheybani F, Sarvghad M, Meshkat Z, Jabbari Nooghabi M (2015). Etiological Diagnosis of Community-Acquired Pneumonia in Adult Patients: A Prospective Hospital-Based Study in Mashhad, Iran. Jundishapur J Microbiol..

[24] Temesgen D, Bereded F, Derbie A, Biadglegne F (2019). Bacteriology of community acquired pneumonia in adult patients at Felege Hiwot Referral Hospital, Northwest Ethiopia: a cross-sectional study. Antimicrob Resist Infect Control..

[25] Wang Y, Kong F, Yang Y, Gilbert GL (2008). A multiplex PCR-based reverse line blot hybridization (mPCR/RLB) assay for detection of bacterial respiratory pathogens in children with pneumonia. Pediatr Pulmonol..

[26] Ghasemshahi S, Ahmadpor M, Booskabadi A, Rezaei H, Poopak B, Hakemi-Vala M (2022). A Six-Month Survey of the Frequency of Extensively Drug-resistant Gram-Negative Bacteria by VITEK 2 System in 2020. Iran J Med Microbiol.

[27] Park HK, Lee S-J, Yoon JW, Shin JW, Shin H-S, Kook J-K (2010). Identification of the cpsA gene as a specific marker for the discrimination of Streptococcus pneumoniae from viridans group streptococci. J Med Microbiol..

[28] Asgari A, Ataee R, Tavana A M, Mirnejad R, Ghorbanalizdgan M (2021). Real-time RT PCR Evaluation of the Xylitol Effect on the Expression of Streptococcus pneumoniae cpsB,cpsD and psaA Genes. Iran J Med Microbiol.

